# EXPLORING GENERATIVITY AMONG CULTURALLY DIVERSE OLDER ADULT VOLUNTEERS

**DOI:** 10.1093/geroni/igae098.3028

**Published:** 2024-12-31

**Authors:** Eireann O’Dea, Andrew Wister, Sarah Canham, Lun Li, Barbara Mitchell

**Affiliations:** Simon Fraser University, Vancouver, British Columbia, Canada; Simon Fraser University, Vancouver, British Columbia, Canada; University of Utah, Salt Lake City, Utah, United States; MacEwan University, Edmonton, Alberta, Canada; Simon Fraser University, Vancouver, British Columbia, Canada

## Abstract

Generativity, the desire to look beyond the self, and teach and guide the next generation, has been described by researchers as an important component of successful aging and a key part of why older adults choose to engage in activities such as volunteering. Recent research on generativity, including Rubinstein et al’s generativity framework (2015), describes how the expression of generativity can be influenced by cultural context, including traditions, sense of heritage, and family relationships. Despite this, so far little research has focused on generative expression among older adults with diverse cultural backgrounds, such as those belonging to ethnocultural minorities. Using Rubinstein’s framework and life history interview as a guide, this study explores generativity among older adult volunteers who belong to ethnocultural minority communities in Vancouver, British Columbia, Canada. 14 participants aged 65 and over participated in in-depth interviews about various life course experiences, in order to bring contextual understanding to their generativity and volunteer activities in later life. Preliminary findings suggest the unique ways in which generative action can develop over the life course, and how it can potentially be influenced by a sense of belonging to an ethnocultural group. Results also speak to the contributions that are made by ethnocultural minority older adults as volunteers in their communities.

